# Type II Abernethy malformation presenting with heart failure: A case report

**DOI:** 10.1016/j.radcr.2025.09.024

**Published:** 2025-09-23

**Authors:** Yanan Gu, Jianjun Luo, Yi Chen, Liangwen Wang, Jiaze Yu, Wei Chen, Yongshi Wang, Zhiping Yan, Xiaolin Wang

**Affiliations:** a Department of Interventional Radiology, Zhongshan Hospital and Shanghai Institute of Medical Imaging, Fudan University, Shanghai, 200032, China; b Department of Echocardiography, Zhongshan Hospital Fudan University, Shanghai, 200032, China

**Keywords:** Congenital extrahepatic portosystemic shunt, Abernethy malformation, Heart failure, Portal vein, Inferior vena cava

## Abstract

Congenital extrahepatic portosystemic shunt (CEPS), known as Abernethy malformation, is a rare vascular anomaly involving aberrant communication between the portal vein (PV) and the inferior vena cava (IVC). In this case, a 51-year-old man with chronic comorbidities presented primarily with symptoms of heart failure rather than hepatic dysfunction. Following endovascular coil embolization of the shunt, the patient's heart failure symptoms improved significantly. This report outlines the diagnostic process and treatment strategy, highlighting the safety and feasibility of a single-session embolization in patients without elevated portal pressure.

## Introduction

Congenital extrahepatic portosystemic shunt (CEPS), also known as Abernethy malformation, is a rare anomaly characterized by a vascular bypass from the portal vein (PV) to the inferior vena cava (IVC) [1]. CEPS manifests with a wide range of clinical presentations, including hepatic nodules, hepatic encephalopathy, hepatopulmonary syndrome, and pulmonary hypertension [1]. It is also frequently associated with cardiac malformations [1].

This case report aims to present an uncommon manifestation of Type II Abernethy malformation primarily as right heart failure, rather than hepatic dysfunction, in a middle-aged adult. By outlining the diagnostic workup, imaging features, and successful 1-step endovascular embolization, we aim to emphasize the importance of considering congenital vascular anomalies in unexplained heart failure and demonstrate the feasibility of interventional treatment in patients with preserved intrahepatic portal flow.

### Case presentation

A 51-year-old man presented with an 11-day history of progressive lower extremity edema and abdominal distension that began 2 days prior. His medical history included hypertension (2 years), type II diabetes mellitus (5 years), and untreated hyperthyroidism (2 years).

Physical examination revealed bilateral scleral icterus, grade II goiter with soft consistency, atrial fibrillation with irregular rhythm, and bilateral pitting edema. Laboratory evaluation revealed evidence of hepatic dysfunction (elevated bilirubin), congestive heart failure (elevated NT-proBNP and troponin), and hyperthyroidism (increased fT3 and fT4) (Table 1).

**Table 1:** Dynamic changes in laboratory parameters before and after coil embolization.

Parameter	Before	After (3-month)	Reference range
PLT (× 10⁹/L)	64	74	125-350
PT (s)	14	12.8	10-30
D-dimer (mg/L)	1.22	0.43	0-0.8
Blood ammonia (μmol/L)	49	27	9-30
Lactic acid (mmol/L)	3.6	1.46	0.7-2.1
NT-proBNP (pg/mL)	529.6	1173	0-100
cTn (ng/L)	28.2	-	0-14
TBil (μmol/L)	81.9	27.6	3.4-20.4
DBil (μmol/L)	22.5	8.2	0-6.8
ALT (U/L)	26	16	9-50
AST (U/L)	42	23	15-40
fT3 (nmol/L)	21	-	3.1-6.8
fT4 (pmol/L)	44.6	-	12-22

ALT, alanine aminotransferase; AST, aspartate aminotransferase; cTn, cardiac troponin; DBIL, direct bilirubin; fT3, free triiodothyronine; fT4, free thyroxine; NT-proBNP, N-terminal pro-brain natriuretic peptide; PLT, platelet count; PT, prothrombin time; TBil, total bilirubin.

**Fig. 1:**
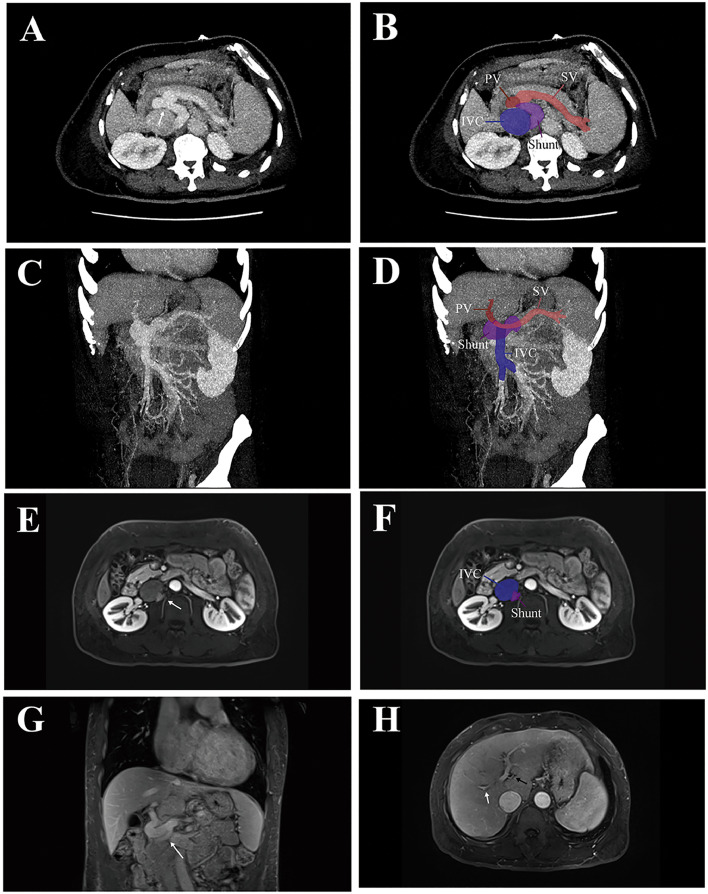
Multimodality imaging examination. (A, B) CTA axial images at the splenic vein (SV, light red) level demonstrate the anomalous tortuous shunt (purple, white arrow in A) connecting the portal vein (PV, dark red) to the inferior vena cava (IVC, blue). (C, D) CTA coronal 3D reconstruction illustrates the course of the shunt (purple) with clear annotation of PV (dark red), SV (light red), and IVC (blue). (E, F) Axial MRI shows the shunt (purple, white arrow) draining into the IVC (blue). (G) Coronal MRI further depicts the twisted, “figure-8″ configuration of the shunt. The maximal diameter of the shunt was approximately 11mm, whereas precise measurement of its length was not feasible due to its tortuous course. (H) Axial MRI demonstrates attenuated intrahepatic PV branches: the left branch (black arrow) and right branch (white arrow).

Echocardiography showed signs of cardiac compensation and mild pulmonary hypertension. Cross-sectional abdominal imaging, computed tomography angiography (CTA) and magnetic resonance imaging (MRI), demonstrated an enlarged IVC and with anomalous contrast inflow on axial views (Figs. 1A-F). Coronal reconstructions revealed a dilated, serpiginous vascular channel draining into the IVC (Fig. 1G), indicating a CEPS consistent with Type II Abernethy malformation. Although intrahepatic PV branches appeared attenuated, they remained visible on MRI (Fig. 1F), suggesting that endovascular occlusion of the shunt was technically feasible.

### Treatment and management

Compared to Type I, Type II Abernethy malformation retains intrahepatic PV branches, making it more amenable to interventional management. In general, endovascular embolization is considered feasible in patients with demonstrable intrahepatic portal flow and low baseline portal pressure. Based on these criteria, a minimally invasive 1-step coil embolization was planned.

**Fig. 2:**
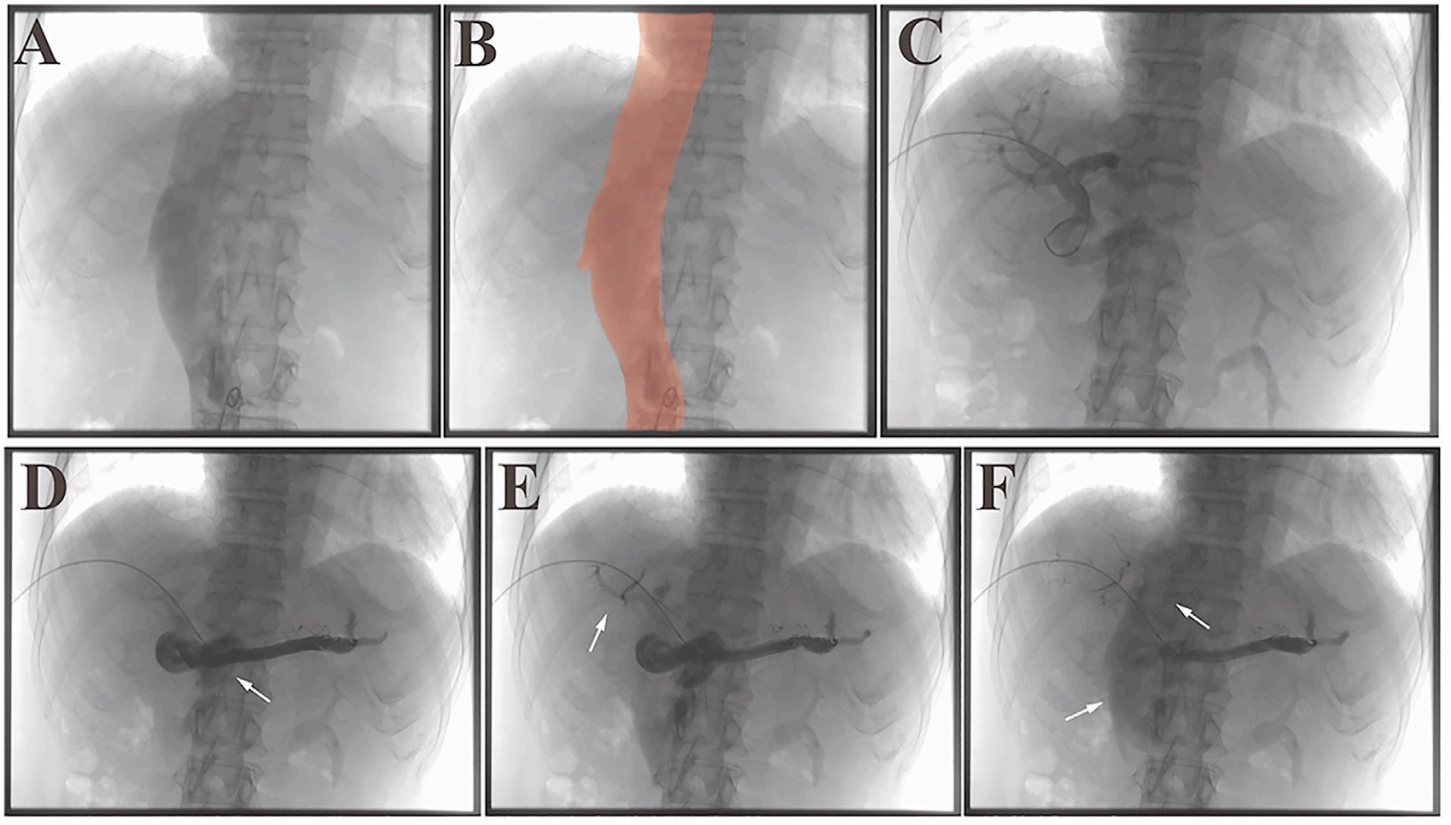
Interventional procedure of detection and imaging. (A, B) Femoral venous access angiography demonstrating the IVC; in B, the IVC is highlighted in red. (C) Ultrasound-guided percutaneous puncture of an intrahepatic portal vein branch shows dilated main portal vein and intrahepatic branches. (D-F) Catheterization of the splenic vein: (D) main portal vein with hepatofugal flow (white arrow), (E) intrahepatic portal branches are attenuated (white arrow) with prominent tortuous collateral vessels, (F) contrast agent refluxing into the IVC (white arrow).

Under local anesthesia, a 4F vascular sheath was introduced via femoral venous access. Angiography revealed high-flow IVC without collateral vessels (Figs. 2A and B), with a distal IVC pressure of 11 mmHg. Initial attempts to access the anomalous vessel via femoral route were unsuccessful. Consequently, ultrasound-guided percutaneous puncture of the right PV was performed. Splenic venography confirmed diversion of hepatic blood flow through tortuous collateral pathways (Figs. 2C-F), with a splenic vein (SV) pressure of 12 mmHg.

**Fig. 3:**
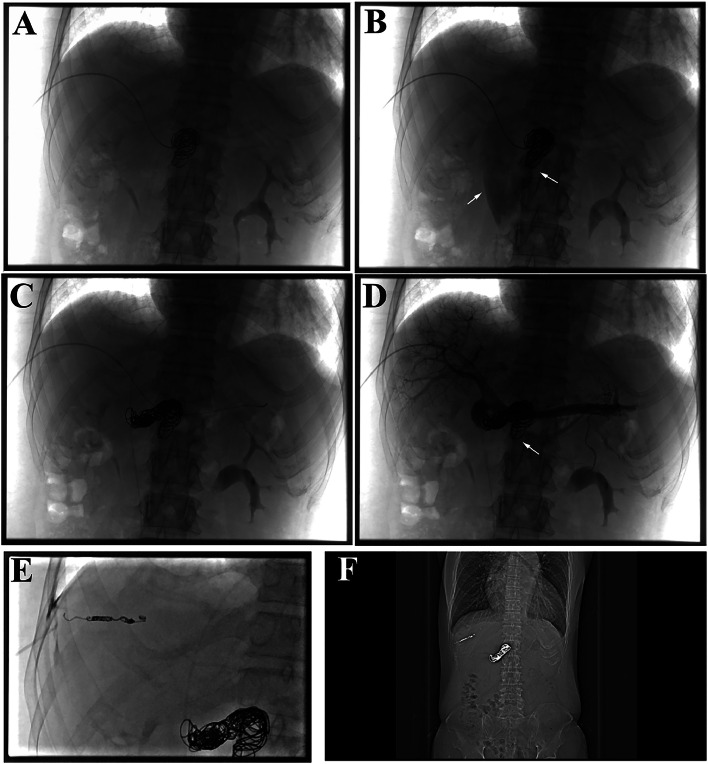
Interventional procedure of embolization and postoperative imagine examination. (A, B) Initial angiography after coil deployment shows incomplete occlusion of the shunt outflow. White arrows indicate the residual shunt outflow. (C, D) Following additional coil embolization, angiography demonstrates complete occlusion of the shunt and improved perfusion of the intrahepatic portal veins. White arrows indicate the prior shunt outflow tract. (E) Coil embolization of the puncture tract is depicted. (F) Follow-up imaging confirms stable coil position.

Embolization began with two 0.035-inch coils (18mm × 40cm) and one 0.035-inch coil (15mm × 40cm). Following superselective microcatheterization, a 0.018-inch coil (22mm × 60cm) was deployed (Fig. 3A). Despite the initial embolization, rapid flow through the abnormal channel persisted (Fig. 3B). Therefore, an additional eight 0.035-inch coils (14mm × 10cm) were inserted (Fig. 3C and D). Post-embolization venography demonstrated restored intrahepatic perfusion (Fig. 3E), with SV pressure increasing to 19 mmHg. Pulmonary artery pressure, measured via femoral catheterization, was 36 mmHg. The puncture tract was sealed with a coil (Fig. 3F).

The patient recovered without complications and was discharged on postoperative day 4. At 3-months follow-up, his symptoms had markedly improved, and laboratory parameters were stable (Table). Long-term follow-up was recommended.

## Discussion

Abernethy malformation is classified into 2 types: Type I, characterized by a complete absence of portal venous flow, and Type II, where partial portal flow is preserved via intrahepatic branches. The latter is often amenable to interventional treatment [1]. While Type II malformations more commonly present with hepatic or pulmonary complications, cardiac manifestations—such as right heart failure—are rare.

In this case, an extrahepatic PSS resulted in excessive venous return, leading to chronic right ventricular volume overload and subsequent remodeling, which might have already been irreversible by the time of occlusion [2]. Moreover, the patient’s comorbidities of hyperthyroidism and atrial fibrillation further complicated the hemodynamics. Hyperthyroidism increases cardiac output and heart rate while reducing systemic vascular resistance, thereby exacerbating heart failure and promoting arrhythmias such as atrial fibrillation [3]. Atrial fibrillation, in turn, contributes to atrial dilation, creating a vicious cycle that amplifies right atrial volume load and structural remodeling [4,5]. This pathophysiology differs from conventional heart failure, which is typically attributed to intrinsic myocardial dysfunction [6]. In our patient, cardiac structure was preserved, but abnormal hemodynamics from the anomalous vascular communication overwhelmed functional capacity. Notably, elevated blood ammonia levels-an indicator of portosystemic shunting-along with mild hepatic enzyme abnormalities, provided important diagnostic clues not typically observed in standard heart failure [7]. Although the patient showed clinical improvement after embolization, with reductions in ammonia and bilirubin levels, NT-proBNP paradoxically increased to 1173 pg/mL at the 3-month follow-up. This may reflect persistent or delayed right ventricular remodeling, ongoing pulmonary hypertension, or continued metabolic stress due to untreated hyperthyroidism [8,9]. As NT-proBNP levels are sensitive to both hemodynamic load and metabolic disturbances, careful longitudinal monitoring is essential to evaluate long-term cardiac recovery.

This metabolic disturbance, along with subtle hepatic enzyme abnormalities, may serve as diagnostic clues and prompt evaluation for underlying vascular anomalies. Imaging plays a pivotal role in diagnosis. Direct visualization of the aberrant vascular pathway linking the PV and systemic circulation on CTA or MRI can confirm the diagnosis, define the shunt anatomy, and differentiate CEPS from other causes of right heart failure [1]. Clinical suspicion should be heightened in patients with unexplained heart failure or pulmonary hypertension in the absence of structural cardiac disease. Initial Doppler ultrasonography may reveal absent or hyplastic PVs or anomalous hepatic vasculature, warranting further evaluation with cross-sectional imaging [10]. In patients with cardiopulmonary complications, transthoracic echocardiography and, if indicated, right heart catheterization should be performed to assess hemodynamics and guide management [11].

When the shunt is lengthy, there is sufficient space for a vascular occlusion device, making endovascular treatment preferable if the portal pressure remains low [12]. Conversely, when the shunt is short, surgical intervention is typically preferred [12]. Liver transplantation is generally required in Type I Abernethy malformation due to the complete absence of portal venous flow [1]. In our case, 1-step coil embolization was feasible because the overall reflux pressure was not elevated. However, hepatofugal flow prevented adequate opacification of the anomalous vessel during contrast injection via femoral venous access into the IVC. As a result, intrahepatic PV puncture was required to delineate the abnormal communication. Although 1-time occlusion carries a potential risk of acute portal hypertension, it may be considered in carefully selected patients with low portal pressure [13]. In contrast, a staged approach may be safer in patients with elevated portal pressure. Nonetheless, the optimal threshold for portal pressure remains undefined, and further studies are warranted to determine a safe and feasible range for single-stage closure [13].

Transcatheter embolization remains the preferred treatment for Type II Abernethy malformation, especially in symptomatic patients [1]. Coils, plugs, or other occlusive devices are commonly employed to close the shunt and redirect portal blood flow into the liver, often resulting in improvement of cardiopulmonary and hepatic symptoms. However, clinicians should remain vigilant for potential post-procedural complications, such as portal hypertension, liver dysfunction, and hepatic encephalopathy, particularly in patients with hypoplastic intrahepatic PVs. Close post-procedural monitoring is essential.

While intervention is widely accepted in symptomatic patients, management of asymptomatic individuals remains controversial [1,14]. Even after embolization, residual pulmonary hypertension may persist, reflecting the irreversible cardiopulmonary remodeling caused by longstanding shunting [14,15]. As the clinical course of CEPS varies depending on the degree of shunting, further studies are needed to refine timing and treatment strategies, particularly for asymptomatic patients.

## Conclusion

In patients presenting with unexplained acute heart failure, rare vascular abnormalities such as CEPS should be considered when routine cardiac evaluation is inconclusive. Abdominal vascular imaging (CTA or MRI) can facilitate diagnosis. Even in older adults, congenital malformations may contribute to chronic disease progression. For selected patients with low portal pressure, single-session shunt embolization offers a feasible and effective treatment approach.

## Patient consent

Written informed consent was obtained from the patient for publication of this case report and any accompanying images. The patient has reviewed the content and agreed to the publication.
